# Regional septal hinge‐point injury contributes to adverse biventricular interactions in pulmonary hypertension

**DOI:** 10.14814/phy2.13332

**Published:** 2017-07-21

**Authors:** Eva Amalie Nielsen, Kenichi Okumura, Mei Sun, Vibeke E. Hjortdal, Andrew N. Redington, Mark K. Friedberg

**Affiliations:** ^1^ Department of Cardiothoracic and Vascular Surgery Aarhus University Hospital Aarhus Denmark; ^2^ The Labatt Family Heart Center and Department of Paediatrics Hospital for Sick Children Toronto Ontario Canada; ^3^ Present address: Department of Pediatric Cardiology Childrens Hospital of Cincinnati Cincinnati Ohio

**Keywords:** Fibrosis, left ventricular dysfunction, right ventricular pressure load

## Abstract

Death and morbidity in pulmonary arterial hypertension (PAH) are often due to right ventricular (RV) failure and associated left ventricular (LV) dysfunction. We investigated regional myocardial remodeling and function as the basis for adverse ventricular‐ventricular interactions in experimental chronic RV pressure overload. Two distinct animal models were studied: A rabbit model of increased RV pressure‐load through progressive pulmonary artery banding A rat model of monocrotaline (MCT)‐induced pulmonary arterial hypertension (PAH). Regional myocardial function was assessed by speckle‐tracking strain echocardiography and ventricular pressures measured by catheterization before termination. Regional RV and LV myocardium was analyzed for collagen content, apoptosis and pro‐fibrotic signaling gene and protein expression. Although the RV developed more fibrosis than the LV; in both models the LV was substantially affected. In both ventricles, particularly the LV, fibrosis developed predominantly at the septal hinge‐point regions in association with decreased regional and global circumferential strain, reduced global RV and LV function and up‐regulation of regional transforming growth factor‐*β*1 (TGF *β*1) and apoptosis signaling. A group of PAH rats who received the TGF *β* blocker SB431542 showed improved RV function and reduced regional hinge‐point myocardial fibrosis. RV pressure‐loading and PAH lead to biventricular TGF *β*1 signaling, fibrosis and apoptosis, predominantly at the septal hinge‐point regions, in association with regional myocardial dysfunction. This suggests that altered geometry and wall stress lead to adverse RV‐LV interactions through the septal hinge‐points to induce LV fibrosis and dysfunction.

## Introduction

RV pressure‐load from pulmonary arterial hypertension (PAH) or pulmonary outflow obstruction carries high morbidity and mortality (Campo et al. 2011; Roche and Redington 2013). While RV dysfunction is an established driver of outcomes; (Sztrymf et al. 2010; Ryan and Archer 2014), left ventricular (LV) dysfunction is increasingly recognized as an important risk factor for symptoms, exercise intolerance, morbidity and mortality (Tzemos et al. 2009; Alkon et al. 2010). Moreover, LV myocardial dysfunction has recently been found to be a key risk factor for early death in PAH (Kassem et al. 2013).

Emphasis has largely been placed on reduced LV filling as the mechanism underlying LV dysfunction in RV pressure load (Marcus et al. 2001). Yet, our previous studies suggest that LV compromise extends to more profound LV myocardial injury and dysfunction. We recently demonstrated in a rabbit model of pulmonary artery banding (PAB) that isolated increased RV pressure load leads not only to RV, but also LV fibrosis and apoptosis through transforming growth factor (TGF*β*1)–connective tissue growth factor (CTGF, also termed as CCNII) signaling (Apitz et al. 2012; Friedberg et al. 2013), attesting to LV myocardial injury and remodeling.

However, the regional mechanisms underlying LV dysfunction and myocardial injury in RV pressure‐load remain inadequately understood. Several reports suggest that these interactions may involve the septal hinge‐point regions where the RV and LV attach to each other and where septal flattening and altered ventricular geometry lead to increased wall stress (Slinker and Glantz 1986). In children with PAH, we recently showed reduced LV myocardial deformation, especially in the septum and adjacent LV regions (Burkett et al. 2015). Isolated reports in PAH patients have suggested that regional myocardial dysfunction is associated with regional fibrosis (Iles et al. 2015) and late gadolinium magnetic resonance imaging has suggested fibrosis at the RV septal hinge‐point regions; (Shehata et al. 2011) a finding that has been related to advanced disease and poor prognosis (Freed et al. 2012). Although these early data suggest that regional myocardial remodeling and dysfunction may drive LV dysfunction and adverse outcomes, regional LV myocardial dysfunction and injury, especially at the septal hinge‐points remains poorly characterized in RV pressure‐loading.

Accordingly, the objective of this study was to investigate regional LV myocardial remodeling and function in experimental chronic RV pressure overload. We hypothesized that increased RV pressure‐load induces regional fibrosis and dysfunction at the RV and LV septal hinge‐point regions through up‐regulation of TGF*β*1 signaling as a mechanism for LV dysfunction.

## Methods

We investigated two distinct models of RV pressure‐load given the different characteristics of pulmonary stenosis versus PAH; and to address potential pitfalls of individual models. For the monocrotaline model, it has been suggested that monocrotaline can induce myocarditis that may potentially impact results. Although monocrotaline does not induce pulmonary vascular proliferative lesions, the severe RV hypertension is well suited to the aims of this study as we do not address the pulmonary disease. For the PAB model, although both PAB and PAH induce RV hypertension, we recognize that PAB is different from PAH and can induce an adaptive RV response versus the maladaptive response induced by PAH.

### Rabbit pulmonary artery banding model

Twenty 6‐week‐old male New Zealand White rabbits had an adjustable vascular cuff (Access technologies, Skokie, IL) placed around the main pulmonary artery through a left thoracotomy (Apitz et al. 2012). The reservoir‐chamber, allowing stepwise cuff inflation, was tunneled to a subcutaneous position in the thorax. Rabbits were divided into two groups: (1) sham‐operated controls (sham) (*n* = 6) in which the pulmonary artery banding (PAB) device was left un‐inflated, and (2) PAB‐group (*n* = 14) with stepwise PAB inflation over 2‐weeks (to avoid acute RV failure), aiming for systemic RV pressures after the third inflation. PAB inflation was monitored by echocardiography for PAB gradient, septal curvature and RV systolic pressure by tricuspid regurgitation Doppler.

Three‐weeks after the final PAB inflation, rabbits were sedated for the terminal experiment in which right and left ventricular high‐fidelity pressure measurements and echocardiography were performed to assess biventricular global and regional function. Thereafter, animals were sacrificed by cardiectomy during deep anesthesia and tissues harvested for histological and molecular investigations. Anesthesia was induced by 3% isoflurane mixed with 2 L 100% oxygen, and maintained by 2–2.5% isoflurane mixed with 2 L 100% oxygen.

### Rat PAH model

Nineteen 6‐week‐old male Sprague–Dawley rats (Charles River, Senneville, Canada) weighing approximately 200 g at protocol onset were divided into the following groups: (A) sham‐controls (*n* = 5) received a single subcutaneous injection of 0.9% saline (B) PAH rats (*n* = 14) received a single subcutaneous injection of 60 mg/kg monocrotaline to induce severe PAH. (C) To further assess the role of TGF*β* signaling in inducing regional LV myocardial remodeling a 3rd group of rats (*n* = 6) received the TGF*β* blocker SB431542 (500 nmol/kg/day by subcutaneous injection). SB431542 was administered from 2‐weeks after the monocrotaline injection, for a duration of 3‐weeks, until the terminal experiment. Four‐weeks after saline or monocrotaline injection, animals were examined with echocardiography and a high‐fidelity micromanometer catheter while anesthetized with 3% isoflurane. Thereafter, animals were sacrificed by cardiectomy during deep anesthesia and tissues harvested for regional histological and molecular investigations.

### Ethics statement

Experiments were approved by the institutional Animal Ethics Committee of the Hospital for Sick Children (Toronto, ON, Canada, approval references #19717 and #32664), and performed in accordance with the Guiding Principles in the Care and Use of Animals of the American Physiologic Society.

### Echocardiography and assessment of regional myocardial performance

Transthoracic echocardiography was performed with a Vivid 7 or E9 system (GE Healthcare, Wauwatosa, WI) using a 12‐MHz phased array probe during 3% isoflurane anesthesia. Maximum frame rates were 275 frame/second. The myocardial performance index (MPI) was used as a measure of global RV and LV systolic and diastolic function (Tei et al. 1996). M‐mode tricuspid annular systolic excursion (TAPSE) and tissue Doppler velocities (TDI) (systolic velocity (S'), early (E') and late (A') diastolic velocities) at the tricuspid lateral annulus were used as measures of RV longitudinal function. The RV fractional area change (FAC) was calculated as the end‐diastolic – end‐systolic area divided by end‐diastolic RV area (Alkon et al. 2010). The LV end‐diastolic (LV EDD), and end‐systolic (ESD) dimensions, fractional shortening (FS) and ejection fraction (EF) were measured by M‐mode echocardiography from the parasternal short‐axis view at the level of the mitral leaflets tips. The eccentricity index was measured as the ratio of the 2 perpendicular LV minor axes (lateral divided by anterior‐posterior dimensions) measured from 2‐dimensional short‐axis images at the LV papillary muscle level (Ryan et al. 1985).

Gray‐scale 2D images were obtained from the apical four‐chamber view for longitudinal strain. To allow analysis of LV regional myocardial performance, specifically at the septal hinge‐point regions, circumferential strain was assessed from para‐sternal short‐axis views. For offline myocardial strain analysis (EchoPac, version 8.0, GE Healthcare, Wauwatosa, WI), the RV and LV endocardial border were traced from an end‐systolic frame to include the free‐wall and septum. The region of interest was manually adjusted to the wall thickness. Adequate tracking was visually verified and the region of interest corrected as necessary.

### Hemodynamic measurements

Hemodynamic measurements and echocardiography were done on the day of the terminal experiment. Systolic and diastolic RV and LV pressures were measured using a 3F high‐fidelity pressure‐tipped catheter. For analog digital converter we used CD Leycom Sigma 5/DF (Hengelo, The Netherlands) for volumes and Millar PCU‐2000 (Houston, TX) for pressures. The software used was Notocord version 4.2 (Croissy Sur Seine, France).

### Tissue collection

**Figure 1:**
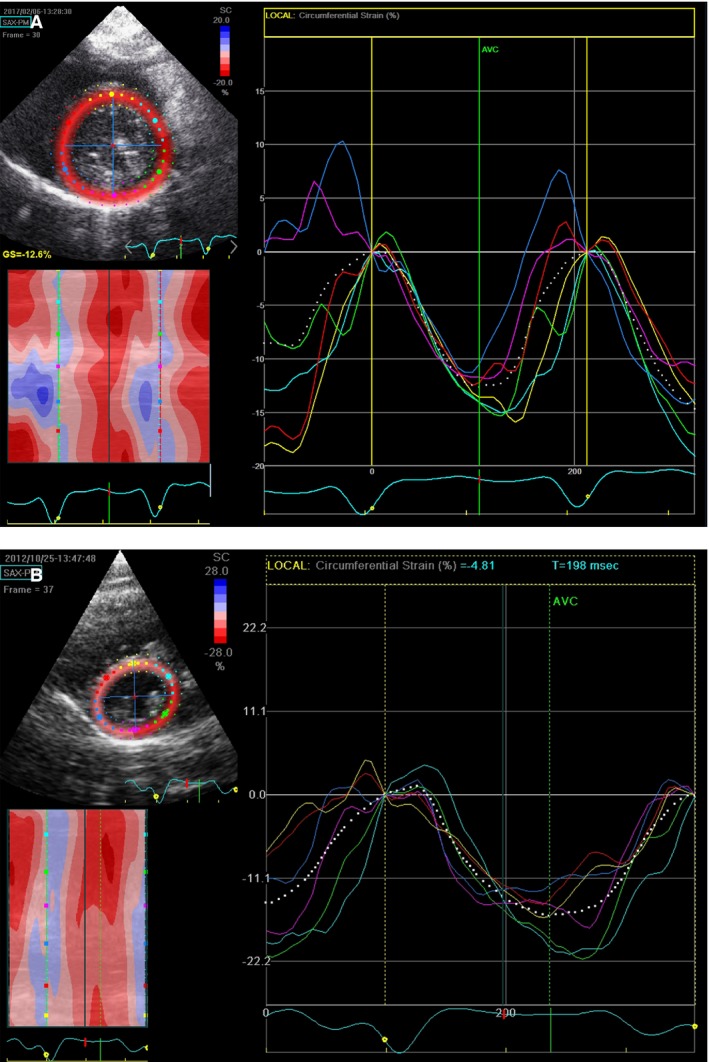
Example of left ventricular (LV) circumferential regional strain in a sham (A) and a PAB (B) rabbit. Depicted are regional LV strain curves. Each strain curve is coded in a color corresponding to the region color‐coded on the short‐axis reference image. Circumferential strain depicts the phasic myocardial shortening (negative portion of the curve) and lengthening (return of the curve to baseline) around the circumference of the LV. The percent shortening is expressed relative to the baseline end‐diastolic length. Note the low global (average of 6‐regional segments) depicted as a white dotted line) LV circumferential strain caused by the low regional strain in the septal, septal hinge‐point and inferior left ventricular regions (cyan, yellow, red and pink curves) in the PAB rabbit.

To assess the regional nature of myocardial remodeling and fibrosis, RV and LV myocardium was sampled from the RV free‐wall, LV free‐wall, septum and RV and LV septal hinge‐point regions as shown in Figure 1A. Tissues were snap frozen and stored at −80° Celsius. Protein analyses by western blotting, and gene expression by real‐time polymerase chain reaction (PCR), were analyzed for each ventricular region. A second sample was preserved in 10% neutral‐buffered formaldehyde and embedded in paraffin for assessment of regional ventricular histology.

### Cardiac morphometry

Fibrosis was assessed as the collagen content; calculated as the regional cardiac collagen volume fraction (ratio of total interstitial collagen area over the sum of total collagen and noncollagen areas in the entire visual field), as quantified by picrosirius red F3BA (PSR) 5 *μ*m stained sections from transverse sections of the entire heart by automated planimetry (Adobe Photoshop CS2, San Jose, CA). Analyses were performed blinded to experimental group.

### Regional pro‐fibrotic signaling and extra‐cellular matrix remodeling

To investigate possible molecular mechanisms of regional LV myocardial fibrosis and remodelling as a correlate for regional myocardial function, western blots and real time PCR were performed in a subgroup of animals where tissues were sectioned in transverse orientation – allowing regional analysis, including the septal hinge‐point regions. The anterior and posterior hinge‐point regions in the rabbits were analyzed together. Given our prior results showing activation of LV pro‐fibrotic molecular signaling secondary to RV pressure‐loading, protein levels of connective tissue growth factor (CTGF), SMAD3, pSMAD3, matrix metalloproteinases‐2 and ‐9 and endothelin‐receptors‐A and B were analyzed as key molecular markers of fibrosis and extra‐cellular matrix remodeling (Leask 2010). *α* and *β* myocyte heavy chain (MHC) protein were analyzed as markers of failing myocardium in response to pressure‐loading (Bogaard et al. 2009a). To investigate apoptosis as a possible cellular mechanism underlying regional LV dysfunction, caspases‐3 and ‐8 were evaluated by western blot as markers of apoptotic activity.

#### Western blot analysis

Cardiac tissues samples were homogenized with lysis buffer and diluted 1:1 with 2 × SDS sample buffer (Invitrogen Novex, Carlsbad, CA). An equal amount of protein (30 *μ*g) was loaded onto each lane of an 8% to 16% Tris‐Glycin gel (Helixx). Proteins were separated by electrophoresis and transferred to a nitrocellulose membrane using an electro‐blotting apparatus (Invitrogen, Carlsbad, CA). Membranes were incubated with 5% Bovine Serum Albumin (BSA) for 1 h to decrease nonspecific binding. Samples were then incubated with the following primary antibodies overnight at 4°C: Connective Tissue Growth Factor (CTGF) (Abcam, Cambridge, UK), SMAD3 and 4 (R&D Systems Inc. Minneapolis, MN), pSMAD3 (Cell Signaling Techonology Inc. Danvers, MA) Endothelin‐1 (Abcam, Cambridge, UK), and matrix metalloproteinases 2 (MMP‐2) (EMD Millipore, Billerica, MA). Caspase 3 and 8 (Cell Signaling Techonology Inc. Danvers, MA), *α* and *β* MHC (EMD Millipore, Billerica, MA) samples were washed and incubated with peroxidase – conjugated secondary antibody, and detected using the Amersham ECL (GE life sciences, Buckinghamshire, UK) detection kit. Glyceraldehyde 3‐phosphate dehydrogenase (GAPDH) was used as the internal standard.

#### Real‐time (rt)‐PCR analysis

Cardiac pro‐fibrotic gene expression levels were measured by the StepOnePlus™ real‐time PCR system (Life Technologies, Carlsbad, CA) using Power SYBR^®^ Green PCR Master Mix (Life Technologies, Carlsbad, CA). Real‐time PCR was performed as follows: 10 min at 95°C, 40 cycles of 95°C for 15 sec, followed by 60°C for 1 min. GAPDH was used as internal standard. Primer sequences are listed in Table 1 of Appendix S1. Cycle threshold values (*C*_t_) were provided by the software, and the 2^−ΔΔCt^ method of relative quantification utilized to compute relative expression levels.

### Statistics

**Figure 2:**
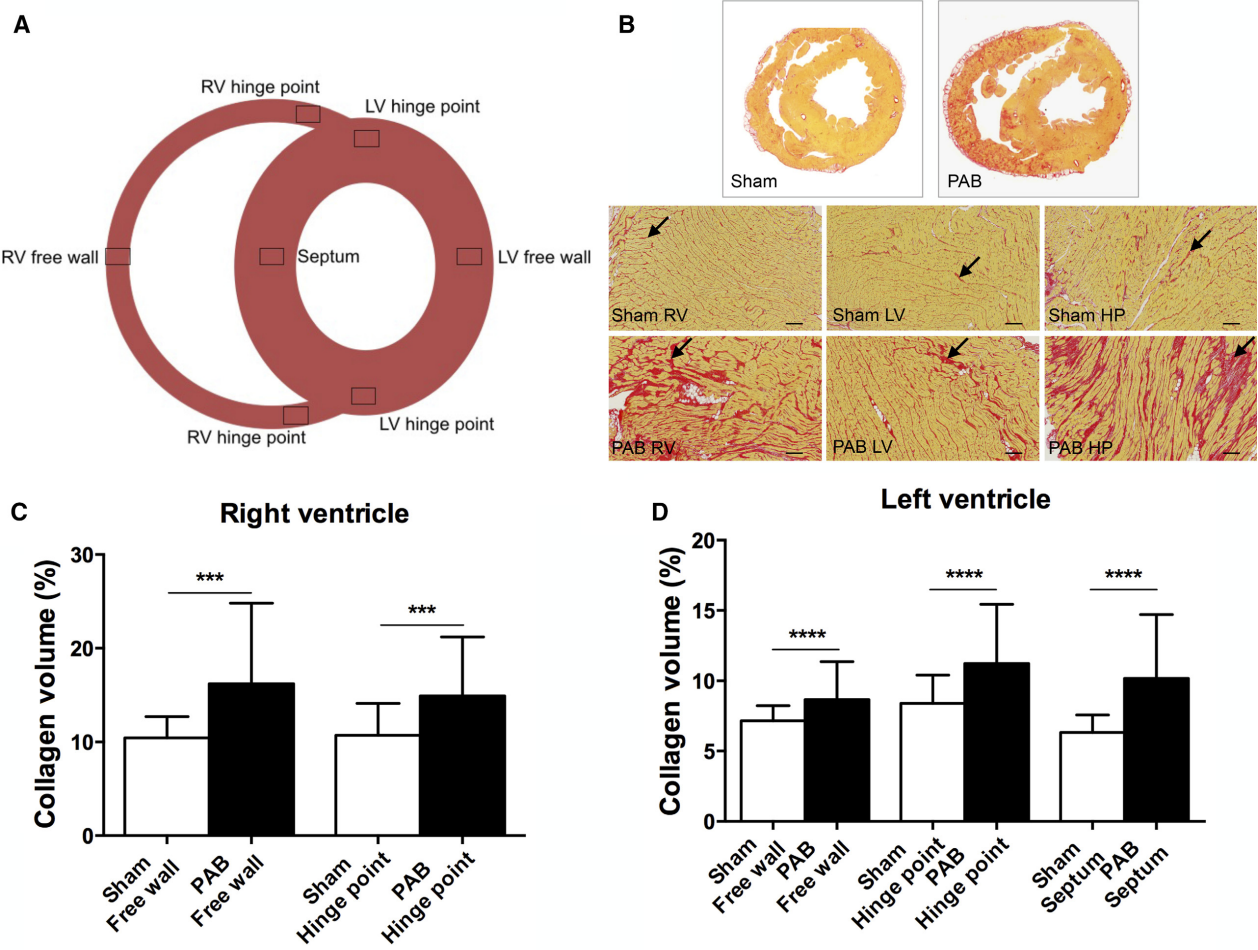
Fibrosis assessed as collagen content percent of the total myocardium in PAB rabbits. (A) Short‐axis diagram depicting the cardiac regions analyzed for collagen content. (B) Picro‐sirius red stained histologic samples of the right and left ventricular free walls. Scale bars: 100 *μ*m, magnification ×100. Higher collagen deposition, indicated by red staining (arrows), is apparent in the right ventricle of PAB animals. Panels (C and D) show right and left ventricular collagen content quantification. (C) There is significantly more fibrosis in the right ventricular free wall and hinge‐point region compared to shams, *P < 0.001. (D) There is significantly more left ventricular regional fibrosis in the free wall, hinge‐point regions and septum compared to shams, *P < 0.0001. The hinge point regions from the anterior and posterior region are analyzed together in each ventricle. Data are presented as mean (SD), Sham: *n* = 6, PAB:* n* = 14. RV, right ventricle and LV, left ventricle.

**Figure 3:**
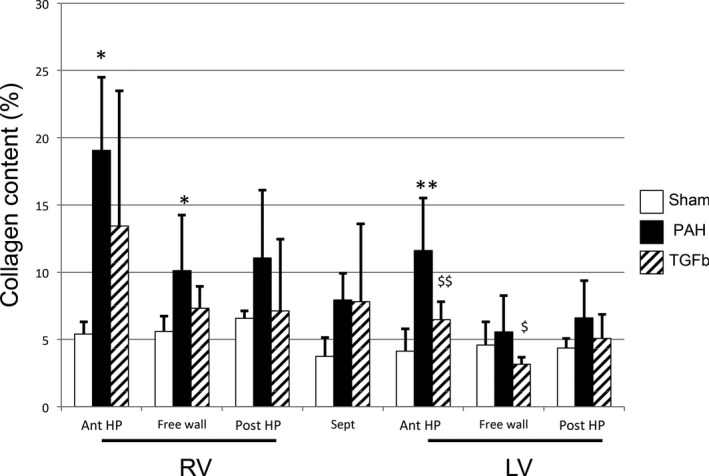
Fibrosis assessed as percent collagen content of the total myocardium in PAH‐ rats. There is significantly more fibrosis in the right ventricular free wall and anterior hinge‐point regions in PAH rats compared with shams. Fibrosis in the LV anterior hinge‐point region in PAH‐rats is higher than shams. *P < 0.05, **P < 0.01. The TGF *β*‐blocker SB431542 reduced fibrosis in the free wall and anterior hinge‐point of the LV. ^$^ *P *< 0.05, ^$$^ *P* < 0.01. Data are presented as mean (SD), Sham: *n* = 8, PAH:* n* = 5 and TGF *β*‐ blocker: *n* = 6. RV, right ventricle; LV, left ventricle; ant, anterior; post, posterior; HP, hinge‐point.

**Figure 4:**
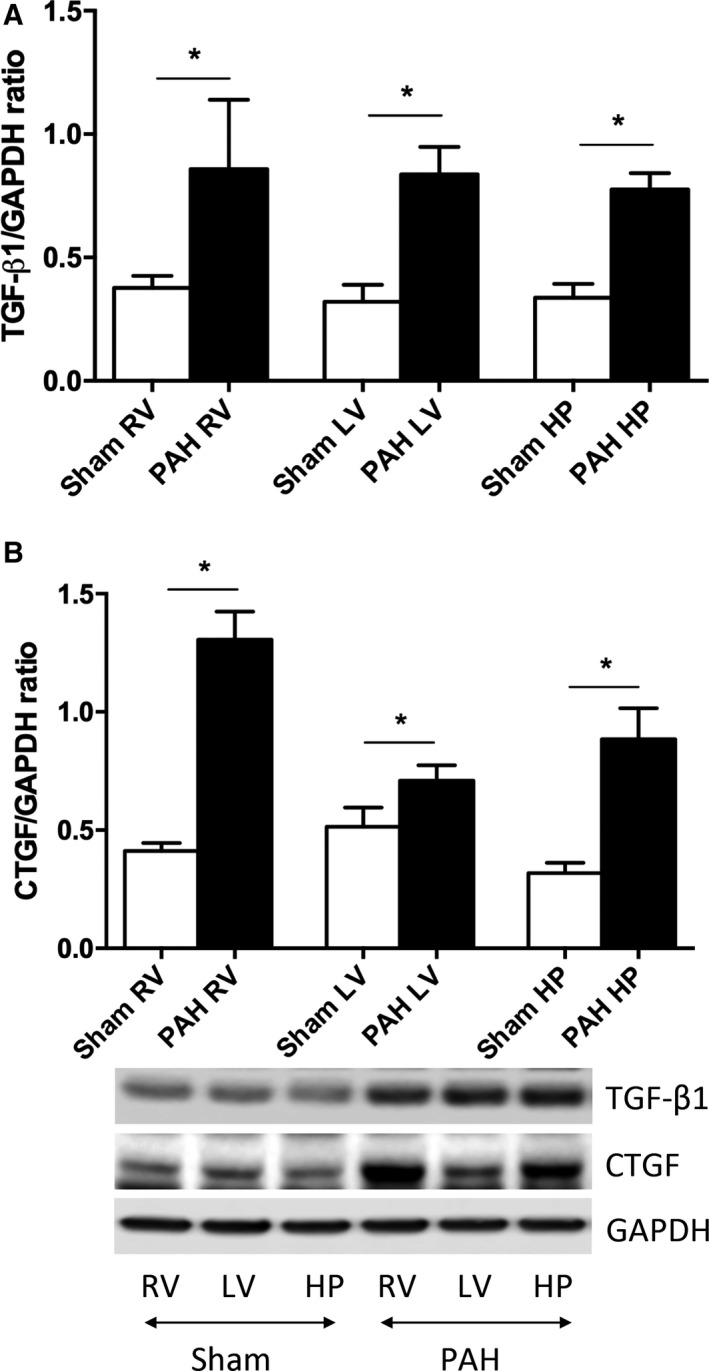
Regional TGF‐*β*1 and CTGF protein levels in PAH rats from the right and left ventricular free wall and septal hinge‐point regions (right and left ventricular septal hinge‐point regions were analyzed together). TGF‐*β*1 protein level increased with PAH in the RV, LV, and the hinge‐point region (HP) compared with shams. CTGF protein level showed the same pattern; the most extensive increase in CTGF in the RV, but also in the LV and hinge‐point region (HP) compared with shams. (Sham: *n* = 4 and PAH:* n* = 4). Data are presented as mean (SD), *P < 0.05. RV, right ventricle and LV, left ventricle.

**Figure 5:**
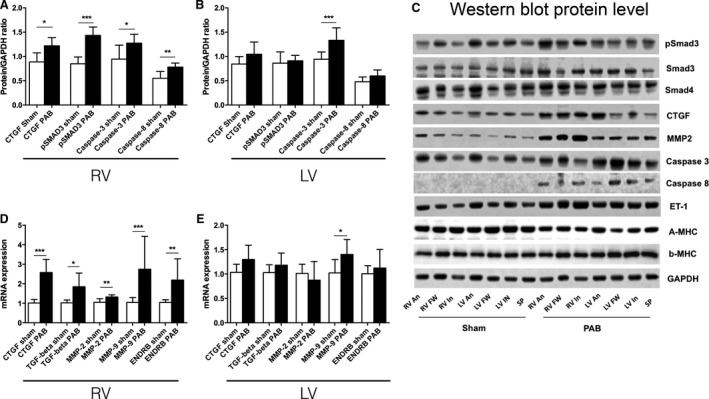
(A and B) Regional right and left ventricular hinge‐point region protein levels (Western blot) in PAB rabbits. Phosphorylated SMAD3 and CTGF, which acts downstream of TGF *β*1, is elevated at the right ventricular hinge‐point regions. The apoptosis related enzymes caspase‐3 and ‐8 in the right ventricular hinge‐point region and caspase‐3 at the left ventricular hinge‐point region are increased compared with shams (Sham: *n* = 5 and PAB:* n *= 3). (C) Western blot gel examples. (D and E) Real time PCR results from the right and left ventricular hinge‐point regions. Gene expression of profibrotic signaling and extracellular matrix remodeling including CTGF, MMP‐2, MMP‐9, and endothelin receptor B (ENDRB) are up‐regulated in the RV hinge point region in PAB rabbits compared with shams, (Sham: *n* = 6 and PAB *n* = 3). Data are presented as mean (SD). *P < 0.05, **P < 0.01, ***P < 0.001 RV, right ventricle and LV, left ventricle.

**Figure 6:**
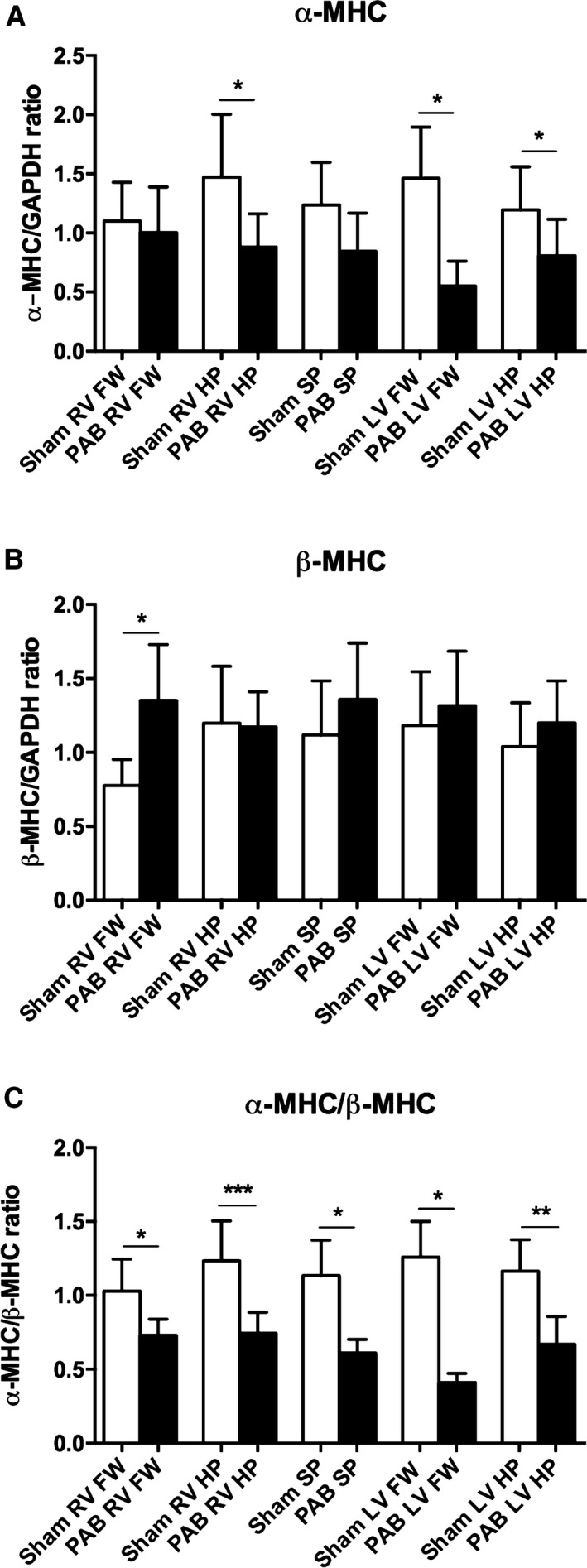
*α* MHC and *β* MHC protein levels assessed by Western blot in tissue samples from rabbits. (A) *α* MHC and B) *β* MHC assessed alone do not reach statistical difference, but there is a shift in the *α* MHC/*β* MHC ratio (decreased *α* MHC and an increase in *β* MHC) at the septal hinge‐point regions of both ventricles in the PAB group. (Sham: *n* = 5 and PAB:* n* = 3) Data are presented as mean (SD), *P < 0.05, **P < 0.01, ***P < 0.001. RV, right ventricle; LV, left ventricle; FW, free wall; SP, septum and HP, hinge‐point.

Grouped data are presented as mean ± standard deviation (SD). Groups (rabbits) were compared using the non‐parametric Mann–Whitney test (Figs. 2, 5, 6, and Tables 1, 2, 3 (rabbits) using GraphPad Prism 6.0 (San Diego, CA). Groups (rats) in Tables 1, 2, 3, and Figures 3 and 4 were compared using ANOVA test using IBM SPSS 19.0. A *P*‐value of less than 0.05 was considered statistically significant. Interclass correlation was assessed by the two‐way random effects‐model using (STATA 12, TX).

**Table 1:** Conductance catheter measurements of pressures in the RV and LV at end‐systole (Pes) and end‐diastole (Ped)

Conductance	Sham rabbits (*n *=* *3)	PAB (*n *=* *11)	Sham rats (*n *=* *8)	PAH (*n *=* *5)	TGF*β*‐blocker (*n *=* *6)
RV Pes (mmHg)	16 (3)	27 (5)**	23 (2)	71 (13)§§	65 (12)§
LV Pes (mmHg)	55 (19)	48 (13)	78 (14)	77 (15)	65 (8)
RV Ped (mmHg)	3 (3)	7 (4)	2 (1)	4 (2)	3 (2)
LV Ped (mmHg)	11 (4)	9 (8)	4 (1)	6 (7)	4 (2)

Results are presented as mean (SD). Sham rabbits versus PAB rabbits: **P <* *0.01. Sham rats versus TGFβ blocker rats: ^§^ *P *<* *0.01, Sham rats versus PAH rats: ^§§^ *P *<* *0.01, Sham rats versus. TGFβ blocker rats: ^§§§^ *P *<* *0.001. PAB, Pulmonary artery banding; PAH, pulmonary arterial hypertension.

**Table 2:** Echocardiography results from both ventricles by 2D measurements, and m‐mode

Echocardiography	Sham rabbits (*n* = 6)	PAB (*n* = 14)	Sham rats (*n* = 8)	PAH (*n* = 5)	TGF*β*‐blocker (*n* = 6)
2D measurements					
LV end‐systolic eccentricity index	1.17 (0.24)	1.21 (0.23)	1.11 (0.04)	2.12 (0.59)§	2.43 (0.84)∧
LV end‐diastolic eccentricity. Index	1.15 (0.26)	1.24 (0.29)	1.08 (0.02)	1.92 (0.33)§§	1.75 (0.43)∧
RV‐FAC (%)	35 (13)	34 (17)	43 (3.2)	21.7 (5.3)§§§	40.7 (7.4)###
M mode					
LV EDD (cm)	1.3 (0.14)	1.1 (0.18)	0.84 (0.1)	0.57 (0.11)§	0.46 (0.08)#
LV ES (cm)	0.9 (0.12)	0.8 (0.15)*	0.55 (0.1)	0.38 (0.09)§§	0.3 (0.07)
LV FS (%)	30 (6)	31 (9)	38 (10)	33 (8)	38 (9)
LV EF (%)	61 (10)	62 (14)	68.7 (8.5)	62.2 (10.7)	73 (11)
TAPSE (cm)	0.4 (0.09)	0.3 (0.08)*	0.26 (0.03)	0.14 (0.05)§§	0.22 (0.07)##
PA band gradient	4 (1)	19 (15)**	—	—	—

Results are presented as mean (SD). Sham rabbits versus PAB rabbits: *P <* *0.05, **P <* *0.01. Sham rats versus PAH rats: ^§^ *P *<* *0.05, ^§§^ *P *<* *0.01, and ^§§§^ *P *<* *0.0001. PAH versus TGFβ ^#^ *P *<* *0.05, ^##^ *P *<* *0.01, ^###^ *P *<* *0.001. Sham rats versus TGFβ ^∧^ *P *<* *0.01. PAB, Pulmonary artery banding; PAH, pulmonary arterial hypertension; ecc, eccentricity; EDD, end‐diastolic diameter; ES, end‐systolic diameter; FS, fraction shortening; EF, ejection fraction, TAPSE, tricuspid annular plane systolic excursion.

**Table 3:** Regional and global strain results

[Median (range)] or [Mean, (SD)]	Sham rabbits (*n* = 6)	PAB (*n* = 14)	Sham rats (*n* = 8)	PAH (*n* = 5)	TGF*β*‐blocker (*n* = 6)
RV longitudinal strain					
Basal RV	−17.0 (6.3)	−10.0 (4.7)*	−17.7 (6.1)	−13.4 (4.2)§	−17 (5.2)
Mid RV	−21.6 (6.9)	−12.5 (5.6)*	−17.1 (5)	−10.7 (5.5)§§	−17.9 (5.2)##
Apical RV	−19.0 (5.5)	−10.0 (4.7)*	−14.2 (4.3)	−8.3 (7.7)	−17.5 (9.6)##
Mean RV	−19.2 (5.1)	−10.8 (4.3)**	−16.4 (3.5)	−10.8 (4.9)§§	−17.5 (6)##
LV circumferential strain					
Ant Septum LV	−10.7 (5.6)	−9.8 (5.2)	−19.7 (7.7)	−13.8 (5.8)§	−21.4 (4.5)##
Anterior LV	−11.3 (5.4)	−11.3 (5.4)	−19 (7)	−14.2 (6.3)	−24.2 (6.7)##
Lateral LV	−11.3 (4.8)	−12.6 (4.8)	−12.6 (5.5)	−10.9 (7.5)	−22.3 (12.9)##
Posterior LV	−15.8 (4.4)	−15.3 (5.1)	−11.3 (5.7)	−7.9 (5.4)	−13.9 (11.1)
Inferior LV	−17.2 (3.7)	−14.3 (6.4)	−16.6 (5.3)	−9.9 (5.7)§	−15.1 (10)
Inf. Septum LV	−14.2 (3.3)	−13.0 (6.6)	−19.4 (7.1)	−12.7 (6.3)§	−19.2 (7.8)#
Mean LV	−13.5 (2.9)	−12.8 (4.1)	−16.4 (3)	−11.5 (3.4)§	−19.4 (6.3)#

Results are presented as mean (SD). Sham rabbits versus PAB rabbits: *P <* *0.05, **P <* *0.01. Sham rats versus PAH rats: ^§^ *P *<* *0.05, ^§§^ *P *<* *0.01. PAH versus TGF*β*‐blocker ^#^ *P *<* *0.05, ^##^ *P *<* *0.01. LV, left ventricle; RV, right ventricle; PAB, pulmonary artery banding; PAH, pulmonary arterial hypertension; ant, anterior; Inf., Inferior.

## Results

One rabbit was sacrificed before the end of the study period due to wound infection, no other rabbits died during the study. Six PAH rats died before the end of the study period due to RV failure. RV systolic pressures were significantly elevated in PAB rabbits and PAH rats, although they were only 2/3 systemic in PAB rabbits at time of terminal experiments (Table 1).

### Echocardiography

RV global function was worse in PAB rabbits and PAH rats versus controls while TGF*β* blockade improved TAPSE and RV‐FAC (Table 2). LV ejection fraction was unchanged in PAB rabbits and PAH rats (Table 2).

### Regional myocardial performance

Longitudinal RV strain was reduced in PAB rabbits and PAH rats compared to controls (Table 3). Global and regional LV circumferential strain was reduced in PAH‐rats compared with controls (Fig.**** 1), especially at, or adjacent to, the septal hinge‐point regions at the anterior LV septum and inferior LV wall (Table 3).

Interclass correlation was performed on LV circumferential strain in 8 rats. Intraobserver and interobserver agreement were moderate at 0.69 and 0.62 respectively.

### Regional myocardial remodeling

PAB rabbits (Fig. 2) and PAH rats (Fig. 3) developed extensive RV and LV fibrosis compared to shams as assessed by collagen content. In both models collagen content was higher in the RV versus LV free wall (PAB: 16.6% ± 7.2% vs. 8.7% ± 2.2%, *P* = 0.001; PAH: 10.1% ± 4.4 vs. 5.6 ± 2.9). In both models, LV, collagen content was higher at the septal hinge‐point regions versus shams (Figs. 2 and 3). In contrast, LV free wall collagen content was marginally (but statistically significantly) higher in PAB rabbits versus controls (Fig. 2), but was not significantly increased in PAH rats versus controls (Fig. 3). Blocking TGF*β*1 in PAH rats reduced collagen content in the LV free wall and LV anterior hinge‐point (Fig. 3).

Given the regional myocardial dysfunction and increased fibrosis, we explored regional myocardial expression of molecular pro‐fibrotic signaling, ECM remodeling and apoptosis.

In PAH rats pro‐fibrotic signaling (TGF*β* and CTGF) was up‐regulated in the RV and LV free‐walls and septal hinge‐point regions compared to shams (Fig. 4A and B). In PAB rabbits the RV hinge‐point region showed the most changes in profibrotic signaling and ECM remodeling including increased pSMAD3, pSMAD3/SMAD3 ratio (Appendix S1 Results), connective tissue growth factor (CTGF) and endothelin‐receptor‐B (ENDRB) (Fig. 5A–D). In the LV, MMP2 and 9 were regionally up‐regulated at the hinge‐point regions and free wall, while pSMAD3/SMAD3 ratio was reduced in the free wall (Appendix **S1**). For complete detailed Western blot and rtPCR results for each region see Tables 2 and 3 in the “Appendix S1”.

Apoptosis‐related proteins were increased in the RV (caspase‐3 and ‐8) and LV (caspase‐3) septal hinge‐point regions in PAB rabbits versus shams (Fig. 5A–C). The *α*/*β* MHC ratio was reduced in all regions, most extensively at the RV and LV septal hinge‐point regions, in PAB rabbits versus shams (Fig. 6A–C).

## Discussion

Although adverse ventricular‐ventricular interactions leading to LV injury and dysfunction are increasingly recognized as important predictors of poor outcomes in RV pressure loading and PAH, the underlying mechanisms remain inadequately defined (Broberg et al. 2011; Roche and Redington 2013). In this study we demonstrate that adverse RV‐LV interactions in RV pressure‐loading occur in a regional distribution, predominantly involving the septal hinge‐point regions. The main findings of this study are that: 1. While the RV carries a higher fibrosis burden than the LV, the LV is substantially affected. 2. Both ventricles, and particularly the LV, develop fibrosis predominantly at the septal hinge‐points and adjacent regions 3. Increased regional fibrosis is associated with up‐regulation of regional TGF*β*1 signaling 4. Regional fibrosis is associated with regional myocardial dysfunction and global biventricular dysfunction.

Together with the geometrical changes and septal flattening that occurs in RV hypertension, and as seen in this study through increased LV eccentricity index, these findings suggest that adverse ventricular‐ventricular interactions and LV injury are mediated through myocardial injury and dysfunction at the septal hinge‐point regions.

### Septal configuration and generation of regional injury

In severe RV pressure loading, septal mechanics change dramatically with systolic and diastolic septal flattening and leftward bowing. While the hemodynamic implications of prolonged leftward shift on reduced LV filling and the Frank‐Starling mechanism are relatively well described (Marcus et al. 2001, 2008), it is unknown whether these geometrical changes induce more profound myocardial injury. Our findings of increased interstitial fibrosis, apoptosis and extracellular matrix remodeling at the RV and LV septal insertion regions suggest this is the case. While for the purposes of this study, in order to better understand ventricular‐ventricular interactions, we analyzed the RV and LV septal hinge‐points separately; in reality they are contiguous. Hence, the RV and LV join and interact at these regions. Our findings are consistent with, and provide a pathophysiological basis for, findings in human PAH patients where late‐gadolinium enhancement MRI, suggestive of increased fibrosis, has been demonstrated at the septal hinge‐points (Sanz et al. 2007; Shehata et al. 2011). Indeed, fibrosis at the RV and LV septal hinge‐point regions is common in PAH and is related to the severity of RV afterload and to reduced ventricular function (Blyth et al. 2005; McCann et al. 2007; Freed et al. 2012). Our findings are therefore important as fibrosis (or at least its imaging correlate) has been observed as an important predictor of symptoms and survival in PAH (Sanz et al. 2007; Shehata et al. 2011). Although unproven, the regional pattern of injury and dysfunction observed in our study suggests that the altered ventricular and septal geometry may be important in driving regional myocardial injury. We, and others, have shown markedly prolonged RV contraction in PAH, causing leftward septal shift in association with reduced cardiac output, reduced exercise capacity and increased mortality (Duffels et al. 2009; Lurz et al. 2009; Tzemos et al. 2009; Alkon et al. 2010). Thus, prolonged RV contraction and septal displacement reduce LV filling, but also alter ventricular geometry presumably increasing wall stress at the RV and LV septal‐hinge‐points (Slinker and Glantz 1986). In the current study, systolic and diastolic LV eccentricity index were abnormal in PAH rats (who have more severe disease) reflecting leftward septal shift. In both the PAH rats and the PAB rabbit model, this geometric change was associated with biventricular regional fibrosis, which was most prominent at the septal hinge‐point regions.

### Molecular mechanisms of regional injury

Our results demonstrate that adverse geometrical interactions caused by RV hypertension translate into adverse molecular signaling and tissue injury and provide a mechanistic basis for the reduced myocardial performance observed at and adjacent to the septal hinge‐point regions. Collagen accumulates in both ventricles during chronic RV pressure loading (Apitz et al. 2012; Friedberg et al. 2013). We (Apitz et al. 2012; Friedberg et al. 2013), and others (Bogaard et al. 2009b), have previously shown that biventricular fibrosis as a result of isolated RV pressure overload is mediated through biochemical crosstalk involving TGF*β*1, CTGF and ET‐1–signaling. We now show that TGF*β*1 pro‐fibrotic molecular signaling is especially up‐regulated at the RV and LV septal hinge‐point regions with increased pSMAD3, CTGF, and ENDRB expression, increased collagen deposition and up‐regulation of apoptosis‐related proteins. Conversely, blocking TGF*β*1 reversed collagen deposition in these regions, stressing the role of TGF*β*1 signaling in converting regional geometrical interactions to regional myocardial fibrosis and dysfunction. Similarly, the increased expression of the proteolytic extracellular matrix enzymes MMP‐2 and ‐9 at the septal hinge‐point regions is consistent with extracellular matrix remodeling. Up‐regulation of these enzymes has been linked with LV dilation during progressive heart failure (Spinale et al. 1998) and in LV failure (Iwanaga et al. 2002) an imbalance between MMP's and their inhibitors has been suggested as a marker of transition from compensated to decompensated myocardial remodeling.

#### Functional implications

Using strain echocardiography, we demonstrate in these animal models of RV pressure loading, not only the well described reduction in RV longitudinal function (Okumura et al. 2014), but also reduced LV myocardial performance, especially in regions where fibrosis and its signaling were increased‐at and adjacent to the septal hinge‐point regions. Speckle tracking strain imaging is difficult in humans, and even more so in small rodents with high heart rates. Therefore, our results should be interpreted within the limitations of the techniques. Nonetheless, to demonstrate regional myocardial function around the LV circumference, and to investigate the septal hinge‐point regions, circumferential and/or radial strain are necessary as these regions cannot be readily interrogated or differentiated using longitudinal strain. Within these limitations, our findings suggest that the tissue injury and fibrosis described above are associated with regional myocardial dysfunction. These results are consistent with reduced LV longitudinal and circumferential myocardial strain described in adult PAH patients; findings associated with early mortality in this population (Hardegree et al. 2013). While LV strain correlated with RV strain in that study, the causes for reduced LV strain were not delineated. Our results suggest that the geographical proximity and interaction of the RV and LV at their hinge‐points to the septum, and the changed geometry, as demonstrated by the increased LV eccentricity index, lead to regional LV myocardial injury and dysfunction. That these findings were regional and consistent in two distinct models of RV pressure‐loading, and across two species, supports the geometrical “theory” as a driver of regional injury, rather than toxic injury induced by monocrotaline. We further observed a reduced *α*MHC/*β*MHC‐ratio in the PAB group consistent with decreased *α*MHC and increased *β*MHC. Similar shifts in the *α*MHC/*β*MHC ratio have been found in heart failure patients (Lowes et al. 1997). *β*MHC ATPase activity is lower than that of *α*MHC and in vitro studies in muscle fragments containing reduced *α*MHC had less power output than those with normal *α*MHC content (Herron and McDonald 2002). This suggests that the changed *α*MHC/*β*MHC‐ratio may contribute to decreased myocardial performance in our experimental models.

### Study limitations

Echocardiography in the PAB rabbits showed elevated RV pressure load as seen by a flat interventricular septum and a high PA‐band gradient during stepwise PAB increments. While the aim was to achieve systemic RV pressures, at the terminal experiment RV pressures were on average only 2/3 systemic. In general, the PAB model simulates pulmonary stenosis and the “adaptive” characteristics of this model versus PAH models are well described (Bishop et al. 1994; Bogaard et al. 2009b). Nonetheless, extensive fibrosis was evident in the LV in PAB rabbits, as well as PAH rats. The limitations of the monocrotaline PAH model are well known (Gomez‐Arroyo et al. 2012). However, as our objective was not to treat PAH, but rather to study the myocardial response to persistent PAH, the model was well suited to our aims. Likewise, while monocrotaline can potentially cause myocarditis we did not find evidence of such and it is unlikely that it significantly affected results (Okumura et al. 2015). The potential limitations of each model are addressed, at least in part, by the inclusion of both. In rabbits, not all protein outcomes were available, most importantly TGF*β*1. The hinge‐points regions were pooled as one group in order to obtain sufficient tissue for the western blots. However, it has been suggested that regional function at the LV anterior hinge‐point is more severely affected than the posterior hinge‐point region (Shehata et al. 2013). We used relatively low‐frequency transducers for rodents, which may potentially underestimate peak values of echo parameters such as tissue velocities and myocardial strain. Nonetheless, frame rates were high and significant differences were found between groups.

We observed considerable variability in the echo results, which may in part be due to small sample sizes. Nonetheless, most RV parameters were significantly different between PAH/PAB versus shams, lending internal validation and strength to the echo data, despite the variability. Moreover, echo was but one of several readouts including fibrosis (the main outcome of interest) and analysis of molecular signaling. The results of cardiac function obtained by echocardiography correlated with these outcomes.

## Conclusions

In conclusion, RV pressure loading and PAH lead to extensive RV and LV fibrosis and apoptosis in association with up‐regulation of molecular fibrosis signaling most prominently at the RV and LV septal hinge‐points in association with decreased myocardial function in these regions. Taken together, our findings suggest that geometrical changes and/or RV‐LV interactions at the septal hinge‐points up‐regulate TGF*β*1 fibrosis signaling to drive adverse ventricular‐ventricular interactions, which are emerging as an important risk factor for mortality in PAH.

## Conflict of Interest

EAN received financial support for her stay at The Hospital for Sick Children from the Bonnelykke foundation and the travel fund at Aarhus University. None of the authors have a financial relationship with a commercial entity that has an interest in the subject of the presented manuscript or other conflicts of interest to disclose.

## Data Accessibility

## Supporting information




**Appendix S1.** Supplement data Western blot and rtPCR.
