# Supplementary material for: Regional septal hinge‐point injury contributes to adverse biventricular interactions in pulmonary hypertension
Source: Physiol Rep. 2017 Jul 21;5(14):e13332. doi: 10.14814/phy2.13332 (PMC5532479; doi:10.14814/phy2.13332)
Supplement: Supplementary file 1 — Appendix S1. Supplement data Western blot and rtPCR. [file PHY2-5-e13332-s001.doc]

Table 1 PCR primers

| ET-1 | Forward, 5′-ACTTCTGCCACCTGGACATCA-3′  Reverse, 5′-ACGCTGCCCTGGTAGGAAAT-3′ |
| --- | --- |
| ENDRA | Forward, 5′-GCTTCTTGCTGCTCATGG ATTAC-3′  Reverse, 5′-CCGAGGTCATCAGGCTCTTG-3′) |
| ENDRB | Forward, 5′-CTGGCCATTTGGAGCTGAGA-3′  Reverse, 5′-TTTGGAACCCCAATTCCTTTAA-3′ |
| TGF–β | Forward, 5′-AGGGCTACCACGCCAACTT-3′  Reverse, 5′-CCGGGTTGTGCTGGTTGTAC-3′ |
| CTGF | Forward, 5′-CCCTGCGTCTTCGGTGGC-3′  Reverse, 5′-AGGCAGTTGGCTCGCATCAT-3′ |
| MMP-2 | Forward, 5′-AGGACTACGACCGCGACAAG-3′  Reverse, 5′-TGTTGCCCAGGAAGGTGAAG-3′ |
| MMP-9 | Forward, 5′-CTTCCAACTTTGA CAGCGACA-3′  Reverse, 5′-GGAGTGATCCAAGCCCAGTG-3′ |
| SMAD3 | Forward, 5′-CCAAACCTGTCCCCGAATC-3′  Reverse, 5′-AGTAGGAGATGGAGCACCAGAATG-3′ |
| SMAD4 | Forward, 5′-GATGGATACGTGGACCCTTCA-3′  Reverse, 5′-CCTATGTGTAACCTTGCTCTCTCAAT-3′ |
| α MHC | Forward, 5′-AGAAGCACGCAACCGAGAA-3′  Reverse, 5′-TCCTCAGCCTGAAGGTCATCT-3′ |
| β MHC | Forward, 5′-GACGGTGGTGGCCCTGTAC-3′  Reverse, 5′-CCTTGCCTTTGCCCTTCTC-3′ |
| GAPDH | Forward, 5′-AGGCCGTGGGCAAGGT-3′  Reverse, 5′-CCTCGGATG CCTGCTTCA-3′ |

*ET-1: Endothelin-1, ENDRA: endothelin receptor A, ENDRB: endothelin receptor B, TGFβ: transforming growth factor β, CTGF: connective tissue growth factor, MMP: matrix metalloproteinases, MHC: myocyte heavy chain, GAPDH: Glyceraldehyde 3-phosphate dehydrogenase.*

Results

Table 2: Western blot results

| **[Median (range)]** | **Sham (n=5)** | **PAB (n=3)** | ***p value*** |
| --- | --- | --- | --- |
| CTGF/GAPDH |  |  |  |
| RV Free wall | 0.73 (0.17) | 1.26 (0.08) | **0.036** |
| RV Hinge points | 0.88 (0.19) | 1.22 (0.17) | **0.016** |
| Septum | 0.74 (0.16) | 0.64 (0.11) | 0.5 |
| LV Free wall | 0.84 (0.17) | 0.79 (0.11) | 0.679 |
| LV Hinge points | 0.84 (0.15) | 1.05 (0.25) | 0.118 |
| Endothelin-1/GAPDH | |  |  |
| RV Free wall | 0.77 (0.23) | 1.47 (0.53) | 0.071 |
| RV Hinge points | 1.03 (0.30) | 1.66 (0.49) | **0.016** |
| Septum | 0.92 (0.26) | 1.20 (0.28) | 0.393 |
| LV Free wall | 1.00 (0.31) | 1.39 (0.51) | 0.393 |
| LV Hinge points | 1.17 (0.29) | 1.15 (0.39) | 0.677 |
| MMP-2/GAPDH |  |  |  |
| RV Free wall | 0.70 (0.28) | 1.39 (0.23) | **0.036** |
| RV Hinge points | 0.87 (0.29) | 1.41 (0.38) | **0.011** |
| Septum | 0.83 (0.37) | 0.95 (0.24) | 0.571 |
| LV Free wall | 0.76 (0.33) | 0.88 (0.04) | 0.250 |
| LV Hinge points | 0.65 (0.17) | 0.81 (0.10) | **0.042** |
| pSMAD3/GAPDH |  |  |  |
| RV Free wall | 0.78 (0.18) | 1.14 (0.13) | **0.036** |
| RV Hinge points | 0.85 (0.14) | 1.44 (0.17) | **0.0002** |
| Septum | 0.91 (0.18) | 0.94 (0.15) | 0.786 |
| LV Free wall | 0.99 (0.19) | 0.93 (0.11) | 0.500 |
| LV Hinge points | 0.86 (0.23) | 0.91 (0.11) | 0.854 |
| SMAD3/GAPDH |  |  |  |
| RV Free wall | 0.75 (0.17) | 0.90 (0.23) | 0.393 |
| RV Hinge points | 0.92 (0.30) | 1.25 (0.10) | **0.008** |
| Septum | 1.20 (0.41) | 0.77 (0.18) | 0.143 |
| LV Free wall | 0.96 (0.23) | 1.04 (0.14) | 0.571 |
| LV Hinge points | 1.01 (0.23) | 0.96 (0.13) | 0.535 |
| SMAD4/GAPDH |  |  |  |
| RV Free wall | 0.97 (0.15) | 1.44 (0.15) | **0.036** |
| RV Hinge points | 1.29 (0.12) | 1.50 (0.18) | **0.029** |
| Septum | 1.33 (0.19) | 1.01 (0.06) | **0.036** |
| LV Free wall | 0.98 (0.10) | 1.14 (0.10) | 0.107 |
| LV Hinge points | 1.26 (0.23) | 1.21 (0.13) | 0.617 |
| pSMAD3/SMAD3 |  |  |  |
| RV Free wall | 1.04 (0.18) | 1.31 (0.25) | 0.143 |
| RV Hinge points | 0.96 (0.14) | 1.14 (0.08) | **0.003** |
| Septum | 0.80 (0.16) | 1.24 (0.12) | **0.036** |
| LV Free wall | 1.05 (0.06) | 0.90 (0.04) | **0.036** |
| LV Hinge points | 0.87 (0.23) | 0.95 (0.05) | 0.312 |
| αMHC/GAPDH |  |  |  |
| RV Free wall | 1.10 (0.33) | 1.00 (0.38) | 0.500 |
| RV Hinge points | 1.47 (0.53) | 0.88 (0.28) | **0.023** |
| Septum | 1.24 (0.34) | 0.84 (0.32) | 0.357 |
| LV Free wall | 1.46 (0.43) | 0.55 (0.21) | **0.036** |
| LV Hinge points | 1.20 (0.36) | 0.81 (0.31) | **0.031** |
| βMHC/GAPDH |  |  |  |
| RV Free wall | 0.78 (0.18) | 1.35 (0.38) | **0.036** |
| RV Hinge points | 1.12 (0.38) | 1.17 (0.24) | 0.946 |
| Septum | 1,12 (0.37) | 1.36 (0.38) | 0.393 |
| LV Free wall | 1.18 (0.36) | 1.32 (0.37) | 0.393 |
| LV Hinge points | 1.04 (0.30) | 1.20 (0.29) | 0.366 |
| αMHC/βMHC |  |  |  |
| RV Free wall | 1.03 (0.22) | 0.73 (0.11) | **0.036** |
| RV Hinge points | 1.23 (0.27) | 0.74 (0.14) | **0.0002** |
| Septum | 1.13 (0.24) | 0.61 (0.09) | **0.036** |
| LV Free wall | 1.26 (0.24) | 0.41 (0.06) | **0.036** |
| LV Hinge points | 1.16 (0.21) | 0.67 (0.19) | **0.002** |
| Caspase 3/GAPDH |  |  |  |
| RV Free wall | 0.72 (0.19) | 1.17 (0.08) | **0.036** |
| RV Hinge points | 0.95 (0.29) | 1.27 (0.18) | **0.031** |
| Septum | 0.99 (0.22) | 0.87 (0.28) | 0.679 |
| LV Free wall | 0.78 (0.25) | 1.62 (0.25) | **0.036** |
| LV Hinge points | 0.94 (0.15) | 1.33 (0.26) | **0.001** |
| Caspase 8/GAPDH |  |  |  |
| RV Free wall | 0.45 (0.12) | 0.73 (0.17) | 0.071 |
| RV Hinge points | 0.55 (0.14) | 0.78 (0.08) | **0.005** |
| Septum | 0.54 (0.19) | 0.58 (0.19) | 0.571 |
| LV Free wall | 0.52 (0.12) | 0.89 (0.04) | **0.036** |
| LV Hinge points | 0.48 (0.10) | 0.60 (0.12) | 0.073 |

*CTGF: connective tissue growth factor, MMP: matrix metalloproteinase, pSMAD: phosphorylated SMAD, MHC: myocyte heavy chain, GAPDH: Glyceraldehyde-3–phosphate dehydrogenase was used as the internal control.*

Table 3: real time PCR results

| **[Median (range)]** | **Sham (n=6)** | **PAB (n=3)** | ***p value*** |
| --- | --- | --- | --- |
| CTGF |  |  |  |
| RV Free wall | 1.05 (0.13) | 1.42 (0.33) | 0.155 |
| RV Hinge points | 1.01 (0.18) | 2.58 (0.18) | **0.0001** |
| Septum | 1.00 (0.11) | 1.10 (0.23) | 0.512 |
| LV Free wall | 1.05 (0.21) | 1.19 (0.14) | 0.405 |
| LV Hinge points | 1.03 (0.17) | 1.30 (0.17) | 0.097 |
| TGF-β-1 |  |  |  |
| RV Free wall | 1.07 (0.14) | 1.56 (0.34) | **0.024** |
| RV Hinge points | 1.02 (0.15) | 1.85 (0.70) | **0.011** |
| Septum | 1.04 (0.15) | 1.09 (0.14) | 0.845 |
| LV Free wall | 1.06 (0.20) | 0.98 (0.21) | 0.619 |
| LV Hinge points | 1.03 (0.16) | 1.18 (0.16) | 0.259 |
| Endothelin-1 |  |  |  |
| RV Free wall | 1.05 (0.15) | 1.19 (0.34) | 0.548 |
| RV Hinge points | 1.00 (0.16) | 1.47 (0.16) | 0.051 |
| Septum | 0.97 (0.16) | 0.86 (0.16) | 0.405 |
| LV Free wall | 1.03 (0.19) | 0.93 (0.19) | 0.476 |
| LV Hinge points | 1.01 (0.19) | 0.90 (0.16) | 0.769 |
| MMP-2 |  |  |  |
| RV Free wall | 1.04 (0.08) | 1.24 (0.10) | **0.024** |
| RV Hinge points | 1.05 (0.18) | 1.32 (0.11) | **0.005** |
| Septum | 1.02 (0.12) | 1.39 (0.14) | **0.024** |
| LV Free wall | 1.02 (0.10) | 1.47 (0.14) | **0.024** |
| LV Hinge points | 1.01 (0.19) | 0.87 (0.38) | 0.347 |
| MMP-9 |  |  |  |
| RV Free wall | 1.06 (0.21) | 3.38 (1.11) | **0.024** |
| RV Hinge points | 1.05 (0.25) | 2.75 (1.69) | **0.0008** |
| Septum | 1.02 (0.22) | 1.30 (0.35) | 0.238 |
| LV Free wall | 1.03 (0.32) | 1.23 (0.22) | 0.548 |
| LV Hinge points | 1.02 (0.27) | 1.40 (0.31) | **0.019** |
| SMAD3 |  |  |  |
| RV Free wall | 1.09 (0.15) | 1.17 (0.26) | 0.726 |
| RV Hinge points | 1.04 (0.15) | 1.13 (0.24) | 0.259 |
| Septum | 1.04 (0.20) | ---- | --- |
| LV Free wall | 1.03 (0.12) | 0.99 (0.28) | 0.905 |
| LV Hinge points | 1.00 (0.14) | 1.07 (0.33) | 0.371 |
| SMAD4 |  |  |  |
| RV Free wall | 1.02 (0.13) | 1.56 (0.43) | 0.095 |
| RV Hinge points | 0.99 (0.18) | 1.52 (0.60) | 0.056 |
| Septum | 1.07 (0.11) | 1.11 (0.31) | 0.714 |
| LV Free wall | 1.05 (0.21) | 1.09 (0.37) | 0.905 |
| LV Hinge points | 1.03 (0.17) | 1.15 (0.35) | 0.492 |
| ENDRA |  |  |  |
| RV Free wall | 1.01 (0.17) | 1.27 (0.57) | 0.548 |
| RV Hinge points | 1.03 (0.16) | 1.25 (0.47) | 0.241 |
| Septum | 1.01 (0.20) | 1.15 (0.46) | 0.548 |
| LV Free wall | 0.99 (0.15) | 1.17 (0.49) | 0.500 |
| LV Hinge points | 1.04 (0.17) | 1.21 (0.39) | 0.241 |
| ENDRB |  |  |  |
| RV Free wall | 1.02 (0.20) | 1.87 (0.52) | **0.024** |
| RV Hinge points | 1.05 (0.13) | 2.19 (1.08) | **0.003** |
| Septum | 1.00 (0.20) | 1.19 (0.47) | 0.548 |
| LV Free wall | 1.04 (0.12) | 1.14 (0.35) | 0.548 |
| LV Hinge points | 1.01 (0.16) | 1.12 (0.38) | 0.348 |
| αMHC |  |  |  |
| RV Free wall | 1.06 (0.19) | 0.66 (0.24) | **0.048** |
| RV Hinge points | 1.01 (0.13) | 0.91 (0.27) | **0.049** |
| Septum | 1.01 (0.11) | 0.82 (0.23) | 0.167 |
| LV Free wall | 1.02 (0.17) | 1.13 (0.16) | 0.381 |
| LV Hinge points | 1.00 (0.09) | 0.90 (0.25) | 0.221 |
| βMHC |  |  |  |
| RV Free wall | 1.03 (0.19) | 1.16 (0.20) | 0.381 |
| RV Hinge points | 1.00 (0.13) | 1.08 (0.29) | 0.172 |
| Septum | 1.01 (0.11) | 1.25 (0.28) | 0.095 |
| LV Free wall | 1.04 (0.17) | 0.98 (0.18) | 0.667 |
| LV Hinge points | 1.03 (0.10) | 1.16 (0.24) | 0.451 |

*CTGF: connective tissue growth factor, TGF: transforming growth factor, MMP: matrix metalloproteinase, ENDRA or B: endothelin receptor A or B, MHC: myocyte heavy chain.*
